# Go with the flow…..

**DOI:** 10.1530/VB-22-0019

**Published:** 2022-08-01

**Authors:** Paul H A Quax, Marie-José T H Goumans

**Affiliations:** 1 Vascular Surgery, Leiden University Medical Center, Leiden, The Netherlands; 2 Cell and Chemical Biology, Leiden University Medical Center, Leiden, Netherlands

The vasculature is an amazing network of flexible pipes connecting all organs and provide them with the necessary oxygen and nutrients needed to maintain tissue homeostasis. With a total length in the body able to circle the globe almost twice, blood vessels work as a team with the heart pumping blood through arteries, capillaries and veins. Serving the entire body, the vasculature is an exciting organ important in both health and disease. Therefore, we are very honoured to act as co-Editors in Chief of *Vascular Biology* and are very pleased that we start this journey with an outstanding and enthusiastic new editorial board and a remarkably dedicated staff at Bioscientifica which will allow us to move forward with new initiatives to expand the journal’s impact. But not without thanking the previous Editor in Chief, Prof Paolo Madeddu, who played an important role in launching *Vascular Biology* as a new journal.

Even to this day, there is still a lot to unravel in how blood vessels function and what the impact of disease is – an exciting field of research. Insights into how cardiovascular cells read biochemical and biomechanical signals and influence their neighbouring cells and function of the vascular system is also an emerging aspect. To date, we realize that not all vessels will behave the same and not even all endothelial cells within one vascular bed will behave similarly. Therefore, advanced cellular and animal models are crucial to fully understand the biology of the vessel and draw the roadmap on how to move from bench to bed.

The scope of the journal will be on broad aspects of vascular biology. These include, amongst others, single cell biology, the immune system, mechanobiology and signalling between the different cell types present within the (cardio)vascular system, which all are crucial players in the field, and also vascular (re)modelling, regeneration, calcification, animal models and alternative models, imaging technologies, biochemical and biomechanics cues affecting (cardio)vascular cells and organs.

The principal mission of *Vascular Biology* is to support investigators engaged in high-impact and high-quality science in vascular biology and disease, through rigorous, fast, careful and fair peer review. This creates high value to the authors, the readers and cardiovascular scientists. *Vascular Biology* has focused on outstanding reviews from leaders in the field with occasional original scientific work. We now would like to put more focus on original scientific work, along with state-of-the-art reviews. Several new and exciting opportunities are planned for the coming months. We will solicit reviews or thematic issues on new science and technologies applied to vascular biology, such as those in imaging, physical sciences, engineering, cell biology, artificial intelligence and computer modelling.

The focus on journals as an arbiter of high-quality science has been magnified by the current culture of immediate information via social media to the lay community. Therefore, we, will with Bioscientifica, regularly communicate the findings of *Vascular Biology* papers through multiple channels. We can be certain that there will be challenges and opportunities along the way, some of which are ongoing while others will occur inevitably in the future. Current challenges are the expansion of new journals including an increasing number of online-only journals and the increasing number of predatory journals and submissions via paper mills, a big threat to academic integrity. These challenges are balanced by new opportunities to identify exciting new research through online preprint archives. *Vascular Biology* will continue to focus on new opportunities as we move the journal forward. This will be done in a manner that not only preserves the roots and maintains the focus of *Vascular Biology* but also keeps an eye towards new science and technology opportunities. In this manner, we can best support the needs of both the current and future generation of scientists and clinicians focused on vascular biology and disease.

We encourage the researchers in the cardiovascular field to submit their manuscripts and look forward to making *Vascular Biology* a well respected journal and a home for this field.

Prof Dr Marie-Jose Goumans

Prof Dr Paul Quax

Co-Editors-in-Chief

